# Dislocation of the Shoulder

**Published:** 1907-12-21

**Authors:** 


### Points in Surgery.

### DISLOCATION OF THE SHOULDER.

The shoulder is a joint whose anatomical structure is such as to allow of the greatest possible freedom of movement, and as such it is an exeremely successful mechanical apparatus. Two of the important factors which go to produce this freedom are the shallowness of the glenoid fossa and the large articular surface of the head of the humerus; only part of this articular surface can be in contact with the scapula in any given position. Stability has been sacrificed to free mobility, and it follows that the joint is particularly liable to the results of strains and violence. This is well demonstrated by the frequency of dislocation of this joint. What power of resistance it has to violence, it owes to the muscles which surround it. Thus in front it is guarded by the subscapularis muscle, and behind by the three muscles attached to the great trochanter. Displacement upwards is prevented by the projection of the acromion process and the strong coraco-acromial ligament, but below it has no muscular support, and the capsule itself is weak and loose, to allow of complete abduction of the arm from the side. When any severe strain is put on this part of the joint, dislocation is the common result. It is generally caused by falling on the arm in the abducted position, as in a patient who falls off the steps of a 'bus. He naturally puts out his arm to save his face from contact with the ground, and this action exposes the weakest part of the joint to the shock. Less commonly, dislocation occurs as the result of a blow in the deltoid region when the arm is held in the position of abduction.

### The Diagnosis.

The picture presented by a patient with a dislocated shoulder is so typical that, when once seen, the nature of the injury can be diagnosed almost at sight. He holds the forearm of the affected side with the other hand; the shoulder is immovable, and the elbow is kept away from the side. The affected shoulder is somewhat depressed, and the sound one is slightly raised. On examination it will be found that the protuberance of the deltoid is flattened, and the tips of the fingers can be inserted under the overhanging edge of the acromion process. Hence Hamilton's test for dislocation of the shoulder, that a straight ruler can be applied at the same time to the acromion and to the external condyle. In a normal arm this is impossible. Other tests are Dugas'—when the hand is placed on the opposite shoulder, the elbow cannot be made to touch the side; and Callaway's—the vertical measurement round the axilla is increased on the damaged side. The head of the humerus is found to occupy an abnormal position. In nearly all cases it occupies a position just below and external to the coracoid process. But the first displacement which occurs is a directly downwards one (subglenoid), and the subcoracoid position is secondary. In certain rare cases the head of the bone goes backwards from the subglenoid position, and comes to lie under the spine of the scapula (subspinous).

### Reduction by Kocher's Method.

The dislocation must be reduced as soon as possible. Trial must first be made of Kocher's method. It is better to have the patient lying on a couch for this than to attempt it sitting up in a chair, as is often done, because the muscles which resist reduction are more relaxed in this position. The manipulations are as follows: externally rotate the arm, adduct and then circumduct the hand on to the opposite shoulder. During the whole manœuvre steady downward traction must be kept on the elbow, in order to keep the capsule tense, and thus facilitate the re-entry of the head of the bone. If this method fails to effect reduction, it is probably due to the fact that some resisting structure lies across the rent in the capsule, and it is wise to give the patient an anæsthetic, either chloroform or ether. Gas is not sufficient for the purpose, because it does not produce complete muscular relaxation. When the patient is completely anæsthetised, Kocher's method must be tried afresh. If it still fails, an attempt must be made to lever the head of the bone into position with the heel in the axilla. The surgeon must take his boot off the foot of the same side as the damaged shoulder, and sitting by the patient's side facing him must apply the sole of his stockinged foot to the axilla, and then by steady traction on the arm try to lever the head of the bone back into place. This is generally successful.

### Reduction by Other Methods.

White of Manchester has suggested yet a third method, by pulling on the fully-extended arm. He claims that when this is done the head of the bone lies opposite the rent in the capsule, and reduction is often effected when other methods fail. After reduction the arm must be bandaged to the side. How long must it be kept in this position? Long enough to allow the rent in the capsule to close, and yet not long enough to allow the formation of adhesions in the joint. As a general rule it should be passively moved after one week, and then every other day for the second week, being re-bandaged after every movement. After a fortnight it may be kept in a sling, and after four weeks active movement may be begun. This treatment may seem unnecessarily vigorous; but if the after-treatment is slovenly, the dislocation will almost certainly recur. And when a shoulder has been dislocated two or three times, it comes out on the slightest provocation. This is well known to hunting men, among whom the accident is a common one, and many of them who have suffered from a dislocated shoulder wear what is known as a martingale. This consists of a strap which encircles the body, with an armlet attached to it, so that the arm cannot be abducted more than five or six inches from the side.

### Open Operation.

If all the methods described above fail to effect reduction, even after two or, say, three anæsthetics, there is no choice left but to reduce the dislocation by open operation. An incision is made as for excising the joint, any resisting structures are cut or, if important, turned out of the way, and the head of the bone put back into the capsule. Of course the patient may prefer not to undergo an operation, since movements of the arm can be performed on account of the mobility of the scapula on the trunk. If a dislocated shoulder is first seen at a later date than one month after the accident, no attempt should be made to reduce it by manipulation, for fear of damaging important structures, such as the axillary vessels or trunks of the brachial plexus, which are fixed by adhesions in the new position. The choice then lies between leaving things as they are and performing an open operation.

